# Targeted Transfection Using PEGylated Cationic Liposomes Directed Towards P-Selectin Increases siRNA Delivery into Activated Endothelial Cells

**DOI:** 10.3390/pharmaceutics11010047

**Published:** 2019-01-21

**Authors:** Cristina Ana Constantinescu, Elena Valeria Fuior, Daniela Rebleanu, Mariana Deleanu, Viorel Simion, Geanina Voicu, Virginie Escriou, Ileana Manduteanu, Maya Simionescu, Manuela Calin

**Affiliations:** 1 Institute of Cellular Biology and Pathology “Nicolae Simionescu”, Bucharest 050568, Romania; cristina.constantinescu@icbp.ro (C.A.C.); elena.fuior@icbp.ro (E.V.F.); daniela.rebleanu@icbp.ro (D.R.); mariana.deleanu@icbp.ro (M.D.); viorel.simion@icbp.ro (V.S.); geanina.voicu@icbp.ro (G.V.); ileana.manduteanu@icbp.ro (I.M.); maya.simionescu@icbp.ro (M.S.); 2 University of Agronomic Sciences and Veterinary Medicine (UASVM), Faculty of Veterinary Medicine, Bucharest 050097, Romania; 3 University of Agronomic Sciences and Veterinary Medicine (UASVM), Faculty of Biotechnologies, Bucharest 011464, Romania; 4 Centre National de la Recherche Scientifique (CNRS), Unité de Technologies Chimiques et Biologiques pour la Santé (UTCBS) UMR 8258, 75006 Paris, France; virginie.escriou@parisdecartes.fr; 5 Institut National de la Santé et de la Recherche Médicale (INSERM), Unité de Technologies Chimiques et Biologiques pour la Santé (UTCBS) U 1022, 75006 Paris, France; 6 Université Paris Descartes, Sorbonne-Paris-Cité University, Unité de Technologies Chimiques et Biologiques pour la Santé (UTCBS), 75006 Paris, France; 7 Chimie ParisTech, PSL Research University, UTCBS, 75005 Paris, France

**Keywords:** endothelial cells, P-selectin, siRNA, targeted delivery, liposomes, gene silencing

## Abstract

The progress in small-interfering RNA (siRNA) therapeutics depends on the development of suitable nanocarriers to perform specific and effective delivery to dysfunctional cells. In this paper, we questioned whether P-selectin, a cell adhesion molecule specifically expressed on the surface of activated endothelial cells (EC) could be employed as a target for nanotherapeutic intervention. To this purpose, we developed and characterized P-selectin targeted PEGylated cationic liposomes able to efficiently pack siRNA and to function as efficient vectors for siRNA delivery to tumour necrosis factor-α (TNF-α) activated EC. Targeted cationic liposomes were obtained by coupling a peptide with high affinity for P-selectin to a functionalized PEGylated phospholipid inserted in the liposomes’ bilayer (Psel-lipo). As control, scrambled peptide coupled cationic liposomes (Scr-lipo) were used. The lipoplexes obtained by complexation of Psel-lipo with siRNA (Psel-lipo/siRNA) were taken up specifically and at a higher extent by TNF-α activated b.End3 endothelial cells as compared to non-targeted Scr-lipo/siRNA. The Psel-lipo/siRNA delivered with high efficiency siRNA into the cells. The lipoplexes were functional as demonstrated by the down-regulation of the selected gene (GAPDH). The results demonstrate an effective targeted delivery of siRNA into cultured activated endothelial cells using P-selectin directed PEGylated cationic liposomes, which subsequently knock-down the desired gene.

## 1. Introduction

An impairment of the physiological functions of vascular endothelial cells (EC), the inner cell layer of blood vessels walls, plays an important role in the pathology of a myriad of diseases having an inflammatory-associated process, such as cardiovascular diseases and cancer [1]. Therefore, the development of EC-targeted therapeutic intervention has attracted a lot of interest and there are hopes that this approach will lead to the progress in the treatment of many human pathologies. In the recent years, RNA interference (RNAi) emerged as a powerful and widely used method for specific silencing the genes that contribute to the progression and exacerbation of chronic inflammation [2]. Chemically synthesized siRNA (small interfering RNA) sequences employ the endogenous RNAi machinery of the cells for processing before binding to its target messenger RNA (mRNA) and gene-specific degradation of mRNA. By encapsulation in a nanocarrier, siRNA is successfully protected from degradation in biological fluids and its delivery into the target cell is facilitated, where siRNA is able to block the synthesis of a specific protein [3]. To achieve a vectorized drug delivery, the existence of a molecular target that is selectively expressed on the surface of diseased EC is crucial. In response to noxious stimuli, the affected EC become “activated” and overexpress cell adhesion molecules, chemokines and cytokines that control the recruitment of circulating leukocytes into the vessels’ intima [4] leading thus to an inflammatory process. Thus, cell adhesion molecules that are overexpressed on the plasma membrane of activated EC can be used as molecular targets for drug delivery. One of such suitable target is P-selectin (CD62P), that is exposed on the surface of EC, on the one hand, due to a very rapid translocation (within minutes) from the membranes of Weibel–Palade bodies after stimulation with inflammatory mediators such as histamine, thrombin or phorbol esters and, on the other hand, due to de novo synthesis (within hours) induced by cytokines, such as interleukin (IL)-1, tumour necrosis factor (TNF)-α, lipopolysaccharide (LPS), resistin or high glucose conditions [5,6,7,8,9]. Apart from in vitro studies showing the increased expression of P-selectin on the surface of cultured EC activated with various inflammatory stimuli, there are studies done in ApoE-deficient mice showing that P-selectin mRNA expression is positively correlated with the progression of lesion formation [10]. Moreover, P-selectin has been proved as a reliable target for atherosclerotic plaque imaging in ApoE-deficient mice using magnetic resonance imaging (MRI) [11,12,13,14] and positron emission tomography (PET) [15]. Furthermore, immunohistochemical studies revealed an increased expression of P-selectin on the endothelium covering human atherosclerotic lesions [16], on the endothelium of patients with unstable angina [17] and in the vasculature of tumours, including lung, ovarian, lymphoma and breast tumours [18], proposing P-selectin as a suitable target for imaging and therapeutic intervention in humans. In addition, P-selectin (140 kDa) extends approximately 40 nm from the endothelial cell’ surface, is internalized via endosomes to the trans-Golgi network and then recycled to the secretory granules [19,20], a sequence that further strengthens its suitability for intracellular delivery of therapeutic agents into activated EC using nanocarriers. Different types of nanoparticles have been surfaced with ligands (e.g. sLex, PSGL-1, GP Ibα, fucosylated polysaccharide, antibodies, peptides) that bind to P-selectin highly expressed on the surface of activated ECs and, a targeted delivery was documented in vitro and in vivo [18,21,22,23,24,25,26,27]. Also, siRNA was delivered, with various degree of success, to the EC using liposomes targeted to other cell adhesion molecules such as E-selectin and VCAM-1 [28,29,30,31,32].

In this study, we hypothesize that P-selectin targeted cationic liposomes could function as efficient vectors for siRNA delivery to activated EC. Thus, a peptide with high affinity for P-selectin [10] was coupled to the surface of PEGylated liposomes containing the cationic lipid 2-{3-[Bis-(3-amino-propyl)-amino]-propylamino}-*N*-ditetradecyl carbamoyl methyl-acetamide (DMAPAP) combined with 1,2-Dioleoyl-sn-glycero-3-phosphoethanolamine (DOPE) and a functionalized PEG derivative (Mal-PEG-DSPE). The targeted cationic liposomes were further complexed with siRNA at various charge ratios. We report here the potential of P-selectin targeted cationic liposomes to protect siRNA, after lipoplexes formation and to specifically and efficiently deliver siRNA to P-selectin expressing activated endothelial cells (b.End3 cells) in static and flow conditions that simulates physiological situation of blood flow. Moreover, we demonstrate that P-selectin targeted lipoplexes are functional, efficiently down regulating GAPDH mRNA expression in activated EC. To the best of our knowledge this is the first study demonstrating that P-selectin can be used as a target for specific and effective delivery of siRNA to activated EC, that subsequently knock-down the mRNA expression of the target gene.

## 2. Material and Methods

### 2.1. Reagents

Cationic lipid 2-{3-[Bis-(3-amino-propyl)-amino]-propylamino}-*N*-ditetradecyl carbamoyl methyl-acetamide (DMAPAP) was synthesized as previously described [33,34]. The other reagents were obtained from following sources: 1,2-Dioleoyl-sn-glycero-3-phosphoethanolamine (DOPE), 1,2-distearoyl-sn-glycero-3-phosphoethanolamine-N-[maleimide(polyethylene glycol)-2000] (Mal-PEG-DSPE) and 1,2-dipalmitoyl-sn-glycero-3-phosphoethanolamine-N-(lissamine Rhodamine B sulfonyl) (ammonium salt) (Rhodamine-PE) from Avanti Polar Lipids (Alabaster, AL, USA); Amicon centrifugal filter columns with a cut-off of 100 kDa from Millipore (Billerica, MA, USA); Spectra/Por dialysis bags with a cut-off of 500–1000 Da from Spectrum Labs (Spectrum Europe BV, Breda, Netherlands); tris-(2-carboxyethyl) phosphine (TCEP) from ThermoFisher Scientific (Waltham, Massachusetts, USA); peptide with high affinity for P-selectin (H-CKKKKLVSVLDLEPLDAAWL-OH, CL-20), a scrambled peptide (H-CKKKKLLSAVLWDELVDPLA-OH, CA-20) and siRNA specific for mouse GAPDH (sense 5’–CAC UCA AGA UUG UCA GCA ATT–3’ and antisense 5’–UUG CUG ACA AUC UUG AGU GAG–3’) and negative scrambled control siRNA (sense 5’–UUC UCC GAA CGU GUC ACG UTT–3’ and antisense 5’–ACG UGA CAC GUU CGG AGA ATT–3’) were synthesized by GeneCust (Dudelange, Luxembourg); Control siRNA (FITC Conjugate)-A from Santa Cruz Biotech (Santa Cruz, CA, USA); Midori Green Advanced (Nippon Genetics, Tokyo, Japan); M-MLV RT from Invitrogen (Life Technologies, Milan, Italy); Taq DNA Polymerase from Invitrogen (ThermoFisher Scientific, Waltham, Massachusetts, USA); Lipofectamine™ RNAiMAX transfection Reagent and siPORT™ NeoFX™ transfection agent from Invitrogen (Carlsbad, CA, USA); primers used to detect the glyceraldehyde 3-phosphate dehydrogenase mRNA level (GAPDH; NCBI Reference Sequence: NM_008084.3; forward 5’–AGCCTCGTCCCGTAGACAAAAT–3’ and reverse 5’–CCGTGAGTGGAGTCATACTGGA–3’) and β-actin mRNA level (NCBI Reference Sequence: NM_007393.5; forward 5’–GTGACGTTGACATCCGTAAAGA–3’ and reverse 5’–GCCGGACTCATCGTACTCC–3’) were synthesized by Eurogentec (Seraing, Belgium); Dulbecco’s modified Eagle’s medium (DMEM), foetal calf serum (FCS), penicillin G, streptomycin from Gibco BRL (Gaithersburg, MD, USA); cell culture plates were from TPP® (Trasadingen, Switzerland); CD62P (P-Selectin) Monoclonal Antibody (Psel.KO2.3) PerCP-eFluor 710 and corresponding Mouse IgG1 K Isotype Control PerCP-eFluor 710 from eBioscience™ (San Diego, CA, USA); microscope slides and aqueous mounting medium Roti®-Mount FluorCare DAPI from Carl Roth (GmBH, Karsruhe, Germany); 18 mm diameter round cover glass from Denville Scientific (Metuchen, NJ, USA); 3-[4,5-dimethylthiazol-2-yl]-2,5-diphenyltetrazolium bromide (MTT), trifluoroacetic acid (TFA), SYBR Green I and all other chemicals were from Sigma-Aldrich (St Louis, MO, USA). Deionized water (18.2 MΏ-cm) was produced in house using Milli-Q system from Millipore (Watford, UK).

### 2.2. Preparation of P-Selectin Directed Cationic Liposomes

A mixture of DMAPAP, DOPE and a maleimide-functionalized PEGylated DSPE (Mal-PEG-DSPE) in chloroform, combined at a molar ratio of 50:49:1 mol% was dried in a rotary evaporator and deposited as a thin film in a balloon bottle. After hydration with molecular biology grade water to reach the final lipid concentration of 20 mM, the formed multilamellar vesicles were extruded through a polycarbonate membrane with pore sizes of 100 nm using a Mini-Extruder (Avanti Polar Lipids, Alabaster, AL, USA). The P-selectin binding peptide, with the sequence NH_2_-CKKKKLVSVLDLEPLDAAWL-COOH, contains the amino acids sequence “LVSVLDLEPLDAAWL” described by Molenaar et al. [10] to bind with high affinity to P-selectin. To this P-selectin specific peptide sequence, were added, at the NH_2_- terminal end, a linker of four lysine residues to increase peptide solubility in aqueous buffers and a cysteine residue for coupling its thiol group to the maleimide group at the distal end of PEG (Mal-PEG-DSPE) inserted into liposomes, as previously described [35]. As control, a scrambled peptide of P-selectin binding sequence (H-CKKKKLLSAVLWDELVDPLA-OH) was coupled to the surface of cationic liposomes (Scr-lipo). Shortly, the coupling procedure consisted in the following steps. Before coupling, the disulphide bonds of the peptide were dissociated by adding the reducing agent TCEP. After 2 hours, the excess TCEP was removed by dialysis using cellulose ester membrane (cut-off of 500-1000 Da), overnight at 4 °C against the coupling buffer (10mM Na_2_HPO_4_, 10 mM NaH_2_PO_4_, 2 mM EDTA, 30 mM NaCl, pH 6.7). Then, the peptide was added at molar ratio of 2:1 (peptide: maleimide) to the liposomes suspensions and mixed together at room temperature for 18 hours. Finally, to saturate the uncoupled maleimide groups, l-cysteine (1 mM) was added for 30 minutes. As a purification step, the mixture was centrifuged using Amicon centrifugal filter units of 100 kDa in order to separate the liposomes from uncoupled peptide and excess l-cysteine. The amount of peptide coupled at the surface of liposomes was quantified by HPLC employing a UHPLC (Agilent 1290 Infinity, Agilent Technologies Santa Clara, California, USA) consisting of a quaternary pump, diode array detector and autosampler. Chromatographic analysis was performed on a Zorbax Eclipse Plus C18 column (2.1 × 150 mm, 3.5 µm, Agilent USA). The UHPLC operating parameters were: column flow rate: 0.25 ml/min, column temperature: 25 °C, the detector wavelength: 220 nm and the injection volume: 5 µl. The mobile phase consisted of water/TFA 0.1% (A) and acetonitrile/TFA 0.1% (B). The gradient elution was as follows: 0 min. 28% B, 5 min. 60% B, 5.5 min. 60% B, 7 min. 28% B, 8 min. 28% B. Subsequently, the concentration of coupled peptide was determined indirectly by measuring the amount of un-coupled, free peptide as previously described [36].

To fluorescently label the phospholipid bilayers of liposomes, 1 mol% Rhodamine-PE was added as an ethanol solution subsequent to liposomes’ preparation.

### 2.3. Formation of Lipoplexes

Targeted (Psel-lipo/siRNA) or non-targeted (Scr-lipo/siRNA) lipoplexes were formed at different +/− charge ratios (R) of 0.5, 1, 2, 4, 6, 8, 10, 20 and 30 by mixing siRNA with corresponding quantities of cationic liposomes, at room temperature, for 30 minutes. siRNA was suspended in 150 mM NaCl and mixed with an equal volume of liposome suspensions in 150 mM NaCl prepared at the appropriate concentrations. The charge ratios were calculated considering that the cationic lipopolyamine DMAPAP has 3 positive charges per molecule and 1 μg DNA contains 3 nmoles negative charges [37].

### 2.4. Characterization of Lipoplexes

#### 2.4.1. Size and Zeta Potential

The size of the liposomes (Psel-lipo and Scr-lipo) and of the lipoplexes (Psel-lipo/siRNA and Scr-lipo/siRNA) was determined by dynamic light scattering (DLS) method on a Zetasizer Nano ZS (ZEN 3600) (Malvern Instruments, Malvern, UK). The size Standard Operating Procedure (SOP) measured the scattered light intensities at an angle of 173°, using the dispersant (water) viscosity as sample viscosity, at 25 °C. Afterwards, a Universal Dip Cell (ZEN1002) for zeta (ζ) potential measurements was immersed into the sample. The zeta potential was determined by electrophoretic light scattering (ELS), by running five consecutive measurements at 5 Volts with 300 seconds delay between measurements, at 25 °C using the Smoluchowski model. The results were analysed using the build-in Zetasizer Software 7.12 (Malvern Instruments, Malvern, UK).

#### 2.4.2. Gel Retardation Assay

The siRNA complexation efficiency of Psel-liposomes at different charge ratios was visualized by agarose gel electrophoresis in Tris-Acetate-EDTA (TAE) buffer (40 mM Tris–HCl, 1%, acetic acid, 1 mM EDTA), as previously described (Uritu et al. 2015 [38]). To breakdown the lipoplexes and release siRNA, 1% Triton™ X-100 was added. After 20 minutes at room temperature, the samples were loaded in 1.5 % agarose gel stained with Midori Green Advanced using 6x Loading Buffer (0.05% Orange G, 30% glycerol). Electrophoresis was performed at 70 V for 20 minutes and visualized with an UV transilluminator.

### 2.5. Cell Culture

Immortalized mouse brain endothelial cells derived from mouse brain (cerebral cortex) endothelioma (bEnd.3) from ECACC (catalogue nr. 96091929) were cultivated, according to recommendations, in Dulbecco’s Modified Eagle’s Medium containing 10% FBS at 37 °C in a humidified 5% CO_2_ incubator. The cells were checked regularly for *Mycoplasma* contamination, employing two methods: PCR assay using specific primers for different *Mycoplasma* species and a bioluminescent assay by means of a commercially available kit (MycoAlert mycoplasma detection kit from Lonza, Basel, Switzerland).

The expression of P-selectin on the surface of bEnd.3 cells was determined in the absence (quiescent cells) or in the presence of TNF-α-activated cells (4 hours, 10 or 50 ng/ml), by flow cytometry using anti-human/mouse CD62P (P-selectin) PerCP-eFluor® 710 (1 μl/10^5^ cells) and a standard flow cytometry protocol using the Gallios Flow Cytometer (Beckman Coulter, Brea, CA, USA).

### 2.6. Evaluation of Lipoplexes Cytotoxicity

To evaluate the viability of bEnd.3 cells after exposure to lipoplexes, the MTT assay was used. The cells were seeded in 96-well culture plates and after 24 hours the cells were exposed to lipoplexes, formed at various +/− charge ratios (R = 0.5, 1, 2, 4, 6, 8, 10, 20, 30) using four concentrations of siRNA (10, 20, 40 and 100 nM). Forty-eight hours later, the medium was removed and replaced with MTT solution (0.5 mg/ml) in DMEM without Phenol Red. After incubation for 3 hours at 37 °C, the formazan crystals formed intracellularly were solubilized by adding the lysis buffer (0.1 N HCl/isopropanol) and further incubating the cells for 4 hours at 37 °C. Optical absorbance was measured at 570 nm with reference at 690 nm using a microplate reader (Tecan GENios, Groedig, Austria). The experiments were done in triplicate and the results were expressed as percentages relative to untreated cells considered as control.

### 2.7. Uptake of Psel-Lipo/siRNA Lipoplexes by TNF-α Activated Endothelial Cells

#### 2.7.1. Lipoplexes-EC Incubation in Static Conditions

To evaluate the global association (binding + internalization) of lipoplexes (Psel-lipo/siRNA and Scr-lipo/siRNA) with activated EC, the bEnd.3 cells were seeded on round cover glasses in 24-well plates (5 × 10^4^ cells/well). After reaching confluency, the cells were activated with TNF-α (10 ng/ml) for 4 hours and then incubated with Rhodamine-PE-labelled lipoplexes (R+/− = 4, 20 nM siRNA) for 10, 30, 60 and 240 minutes at 37 °C, in an incubator. To investigate the specificity of Psel-lipo/siRNA interaction with activated EC, competitive studies were performed. Before incubation with Psel-lipo/siRNA lipoplexes, the cells, were preincubated for 1 hour with an excess of P-selectin binding peptide (~25-fold higher concentration of peptide as compared to peptide coupled to the Psel-liposomes surface) before incubation with Psel-lipo/siRNA lipoplexes. At the end, following washing with PBS, the glass covers were mounted on microscope slides with Roti®-Mount FluorCare DAPI and the cells were subsequently investigated by fluorescence microscopy (Olympus IX81 microscope). 

To quantify the fluorescent signal from Rhodamine-labelled Psel-lipo/siRNA lipoplexes, the background was subtracted from the micrographs using CellSens Dimension 1.5 software© Olympus Corporation (Shinjuku, Tokyo, Japan) then the mean range of pixels corresponding to each fluorescent signal (red for Rhodamine-PE and blue for DAPI) was determined using the histogram generated by Corel®Photo-PaintTM X8 (Corel Corporation, Ottawa, Canada). For each captured image, the data for the red histogram (Rhodamine-PE labelled lipoplexes) was normalized to the blue histogram (DAPI stained nuclei).

#### 2.7.2. Lipoplexes-EC Incubation in Dynamic Conditions

To mimic the in vivo conditions of interaction between intravenously injected P-selectin targeted lipoplexes and the endothelium, flow chamber experiments using the Focht Chamber System 2 (FCS2®, Bioptechs, Butler, PA, USA) were performed. The system enables real-time microscope observation of the interaction between nanoparticles and cells under laminar flow perfusion with precise temperature control. The bEnd.3 cells were cultured on 40 mm glass coverslips and after 24 hours, the coverslip was rinsed with warm DMEM and placed into the parallel plate flow chamber of FCS2 system, using the 0.5 mm thick silicone gasket with a 14 × 24 mm rectangle cut in the centre and setting the Stable Z System (Bioptechs, Butler, PA, USA) to maintain the temperature of the system at 37 °C. The flow chamber was placed on the stage of a fluorescence microscope (Olympus IX81) and the flow medium consisting of 4 ml of cell culture medium containing Rhodamine-PE-labelled lipoplexes (R+/− = 4, 20 nM siRNA) was re-circulated for 60 minutes at a shear rate of ~3.8 s^−1^ (shear stress ~3.4 dynes/cm^2^) using a perfusion pump. After 10, 30 and 60 minutes of lipoplexes perfusion, phase contrast and fluorescence images were acquired using CellSense Dimensions software. After 60 minutes of incubation in flow conditions, at the end of experiment, the coverslips were removed from the FCS2 system and the cells were mounted with Roti®-Mount FluorCare DAPI. Fluorescence micrographs using TRITC (red) and DAPI (blue) filters were captured.

### 2.8. Intracellular Delivery of siRNA by Psel-Lipo/siRNA Lipoplexes

To investigate the efficacy of siRNA delivery into endothelial cells, FITC-labelled control siRNA was used to form Psel-lipo/siRNA lipoplexes (R+/− = 4, 20 nM siRNA). bEnd.3 cells were seeded in 24-well plates (5 × 10^4^ cells/well) and after 24 hours, the cells were activated for 4 hours with TNF-α (10 ng/ml) and then incubated with medium containing Psel-lipo/siRNA lipoplexes. The commercially available siRNA vectors, Lipofectamine™ RNAiMAX Transfection Reagent (Invitrogen, Carlsbad, CA, USA) and siPORT^TM^ NeoFX (Ambion, ThermoFisher Scientific, Waltham, Massachusetts, USA) were used according to their respective manufacturers’ instructions: 2 µL/well Lipofectamine™ RNAiMAX and 1.67 µL/well siRNA/siPORT^TM^ NeoFX with the same final siRNA concentration of 20 nM. After 4 hours, the cells were rinsed with PBS, detached with 0.25% trypsin/0.53 mM EDTA, pelleted by centrifugation, resuspended in FACS buffer (3% BSA/5 mM EDTA/PBS) and analysed with a Gallios Flow Cytometer (Beckman Coulter), using blue laser excitation at 494 nm and emission at 520 nm in the FL1 channel (35,000 events counted for each sample). The efficiency of intracellular delivery of siRNA was determined as the percentage of FITC-positive cells, gated in FL-1 channel.

### 2.9. Knock-Down of GAPDH mRNA by Specific siRNA Delivered Intracellularly by Psel-Lipo/siRNA

A siRNA sequence targeting the specific coding regions of the mouse glyceraldehyde-3-phosphate dehydrogenase (GAPDH) transcript was delivered into TNF-α activated bEnd.3 cells using Psel-lipo/siRNA-GAPDH and Scr-lipo/siRNA-GAPDH lipoplexes at R = 4, 10, 20 and 30 using 100 nM siRNA. The GAPDH mRNA level was determined by quantitative-PCR at 48 hours after transfection. To validate the specificity of GAPDH down-regulation, an irrelevant siRNA sequence (scrambled) was used as control. The RNA was isolated by precipitation using TRIzol reagent according to the manufacturer’s protocol. Then, RNA was resuspended in RNAse-free water and the RNA concentration of each sample was determined using Nanodrop™ 2000 Spectrophotometer (ThermoFisher Scientific, Waltham, MA, USA). First-strand cDNA was synthesized by reverse transcription using M-MLV RT, starting from 1 μg of total RNA. Gene expression real-time quantitative PCR experiments were performed on a ViiA7 system (Applied Biosystems, Thermo Fisher Scientific) in 384-well plates and data were analysed utilizing the ViiA7 Software v1.2 (Applied Biosystems, ThermoFisher Scientific, Waltham, MA, USA). A cDNA amount equivalent to an initial concentration of 25 ng RNA was used for the amplification reactions. PCR reactions were optimized for a 10 µL volume, with concentrations as following: 1.5 mM MgCl_2_, 0.25 mM dNTP, 1 µM of each of the forward and reverse primers, 0.5 U Taq DNA Polymerase (Invitrogen) and SYBR Green I (SIGMA-Aldrich, St Louis, MO, USA) as a probe (1x). The PCR parameters were 95 °C for 10 min, followed by 40 cycles at 95 °C for 15 s and 60 °C for 1 min. The amplification efficiencies for the primers were 92.6 % and 93.2% for GAPDH and β-actin, respectively. Each sample was measured in triplicate and the expression levels of the silenced gene of interest (GAPDH) were normalized to the expression of the reference gene (β-actin) and results were presented as fold change as compared to the control cells (unsilenced condition), using the 2^−ΔΔCT^ method [39]. Subsequently, the reaction products were visualized by agarose gel electrophoresis and Midori Green stain.

### 2.10. Statistical Analysis

The results were expressed as mean ± S.D. or mean ± S.E.M. and experiments were performed at least in triplicate. Statistical evaluation was carried out by unpaired Student’s t-test (two tailed) using GraphPad Prism 7 software (GraphPad Software, San Diego, CA, USA). Differences were considered to be statistically significant when p < 0.05.

## 3. Results

### 3.1. Characterization of Psel-Lipo/siRNA Lipoplexes

**Figure 1:**
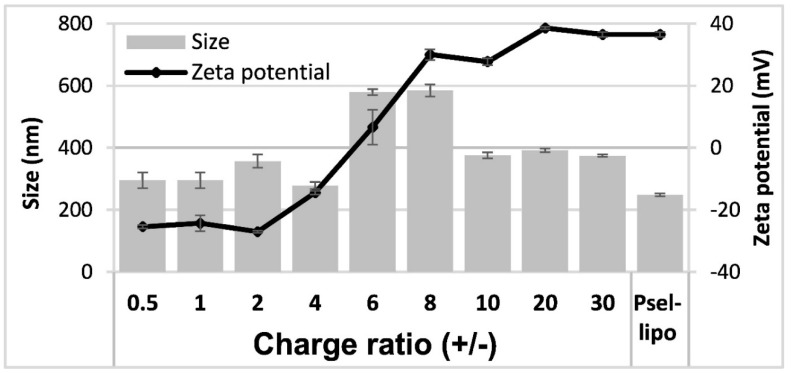
Average hydrodynamic diameter and ζ-potential of P-selectin targeted liposomes (Psel-lipo) and lipoplexes (Psel-lipo/siRNA) at different charge ratios +/− (R) for 100 nM final siRNA concentration. Results are reported as mean ± S.D. for three individual measurements.

PEG-stabilized cationic liposomes consisting of a mixture of a cationic lipid DMAPAP, a helper lipid DOPE and a PEGylated phospholipid used for coupling the peptide with affinity for P-selectin have been prepared. P-selectin binding peptide or a scrambled peptide were covalently coupled via a thioether bond to the distal end of maleimide functionalized PEG-DSPE incorporated into liposomal bilayers. HPLC results showed a coupling efficiency of about 9.5 μg peptide/μmol phospholipid. The average hydrodynamic diameter and ζ-potential of Psel-lipo and Psel-lipo/siRNA lipoplexes formed at different charge ratios +/− were measured after 1:100 dilution in distilled water (Figure 1).

There were no significant differences in the average hydrodynamic diameter between Psel-lipo and Scr-lipo nor between Psel-lipo/siRNA and Scr-lipo/siRNA. In the case of lipoplexes, for the charge ratios up to 4, the size of both type of lipoplexes was similar with that of liposomes (around 300 nm). It was found an increase in the size of lipoplexes was observed as the charge ratios increased, up to 600 nm for ratios of 6 and 8 and ~400 nm for higher ratios, suggesting a dynamic charge interaction. The ζ-potential of liposomes was positive with a value of ~+35 mV for both Psel-lipo and Scr-lipo. For Psel-lipo/siRNA lipoplexes, the ζ-potential was negative with values of around -25 mV at charge ratios of 0.5, 1 and 2 and increased at ~−15 mV (R = 4), ~+5 mV (R = 6), ~+30 mV (R = 8 and R = 10) and ~+35 mV (R = 20 and R = 30).

### 3.2. Psel-Lipo Effectively Packs siRNA

**Figure 2:**
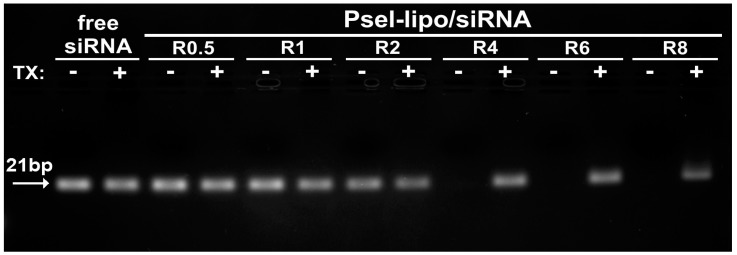
Agarose gel retardation assay performed for free siRNA and Psel-lipo/siRNA complexes at different charge ratios +/− (R) (200 ng siRNA/lane), in the absence or presence of 1% Triton X-100 (TX).

Agarose gel retardation assay was used to evaluate the siRNA loading capacity of lipoplexes and protection of siRNA by cationic Psel-lipo. Free siRNA or electrostatic lipoplexes between Psel-lipo and siRNA, obtained at different +/− charge ratios (0.5, 1, 2, 4, 6 and 8), in the absence or the presence of 1% Triton™ X-100, were loaded in a 1 % agarose gel containing Midori-Green and the siRNA lanes were visualized under UV light (Figure 2). The amount of siRNA was maintained constant at 200 ng/lane. Compared to free siRNA, the migration of siRNA is completely blocked in the case of complexation with Psel-lipo at charge ratios +/− higher or equal to 4, suggesting that starting with R = 4, the siRNA is packed and protected by Psel-lipo. The addition of a surfactant (1% Triton X-100) released the entrapped siRNA that became visible in the gel at any charge ratio (Figure 2).

### 3.3. Lipoplexes Cytotoxicity

**Figure 3:**
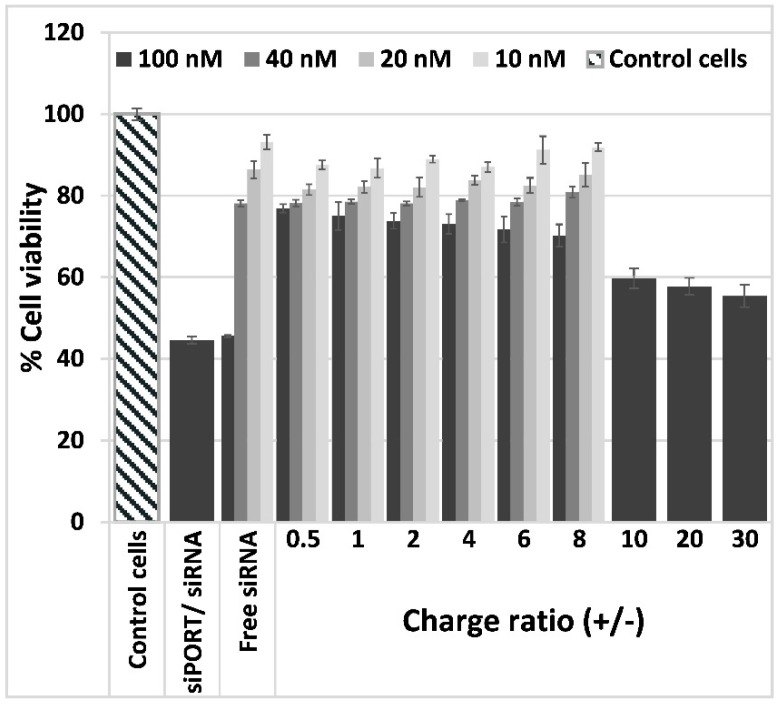
Viability of b.End3 cells exposed for 48 hours to different charge ratios (+/−) of Psel-lipo/siRNA lipoplexes (0.5, 1, 2, 4, 6, 8, 10, 20, 30) formed with different siRNA concentrations (10 nM, 20 nM, 40 nM, 100 nM). Data is presented as mean ± S.E.M. of three identical experiments made in three replicates.

The effect of Psel-lipo/siRNA lipoplexes on EC viability was investigated by MTT assay. The cellular viability of bEnd.3 cells exposed to free siRNA or to lipoplexes at different charge ratios resulting for four different siRNA concentration (10 nM, 20 nM, 40 nM and 100 nM), was determined as percent from control cells cultured in normal medium in the absence of siRNA or lipoplexes, considered to be 100% viable (Figure 3). 

The data showed that the viability of bEnd.3 cells was reduced by incubation with free siRNA as a function of concentration, 100 nM free siRNA inducing the more pronounced decrease in cells viability (~45%). The same level of cellular viability was attained when cells were incubated with commercial transfection vector siPORT^TM^ NeoFX carrying 100 nM siRNA. By contrast, the complexation of 100 nM siRNA with Psel-lipo determined a significantly higher percentage of viability (over 70% viability for charge ratios between 0.5–8 and around 60 % viability for charge ratios 10, 20 and 30). In the case of EC incubation with Psel-lipo/siRNA lipoplexes made using 10, 20 or 40 nM siRNA, the cellular viability was decreased by ~10–20% as compared to control cells, as function of siRNA concentration and lipoplexes charge ratio +/−. No significant difference was obtained in EC viabilities when the cells were incubated in the presence of lipoplexes formed at different charge ratios +/− using various siRNA and incubation with liposomes at the same concentrations as that used to make lipoplexes (data not shown).

### 3.4. Psel-Lipo/siRNA Lipoplexes Are Specifically Taken Up by Activated Endothelial Cells

#### 3.4.1. P-Selectin Is Expressed Predominantly on the Surface of Activated Bend.3 Cells

**Figure 4:**
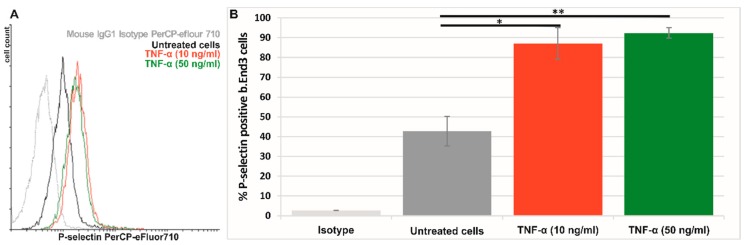
(**A**) Representative flow cytometry chart showing the expression of P-selectin on the surface of quiescent (untreated) (black line) and TNF-α activated bEnd.3 cells (red and green lines). (**B**) Percentages of P-selectin positive cells as determined by flow cytometry experiments and plotted from a single experiment using triplicate probes. Bar graph shows data as mean ± S.E.M (representative experiment made in triplicates). * p < 0.05, ** p < 0.01.

The expression of P-selectin on the surface of quiescent or TNF-α activated bEnd.3 cells was assessed by flow cytometry. In the absence of TNF-α activation, ~40% of bEnd.3 cells express P-selectin (Figure 4). The activation of bEnd.3 cells with TNF-α (10 or 50 ng/ml) for 4 hours caused an approximately two-fold increase in P-selectin expression. It was found that more than 85% of bEnd.3 cells were positive for P-selectin when the cells were exposed to 10 ng/ml TNF-α. No significant difference was detected between activation with 10 ng/ml and 50 ng/ml TNF-α. Therefore, in all further studies, the bEnd.3 cells were activated for 4 hours with 10 ng/ml TNF-α and then used to assess the interaction of Psel-lipo/siRNA or Scr-lipo/siRNA lipoplexes with the cells.

#### 3.4.2. Psel-Lipo/siRNA Lipoplexes Are Specifically Internalized by TNF-α Activated Endothelial Cells

The interaction between Psel-lipo/siRNA and Scr-lipo/siRNA lipoplexes (R+/− = 4; 20 nM siRNA) and the EC was investigated at 37 °C for four-time points (10, 30, 60 and 240 minutes) by fluorescence microscopy, using cationic liposomes labelled with Rhodamine-PE, in static conditions or in conditions that simulate the blood flow.

**Figure 5:**
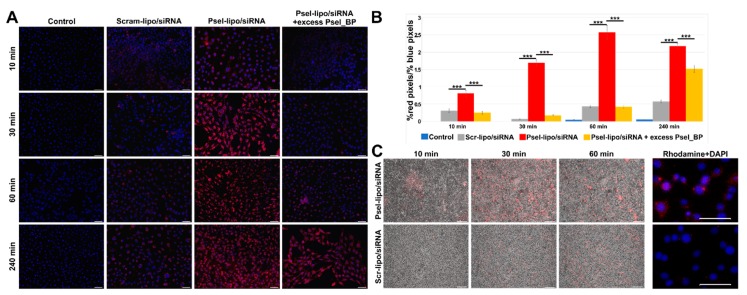
(**A**) Uptake of Rhodamine B-labelled Psel-lipo/siRNA and Scr-lipo/siRNA lipoplexes (charge ratio R +/− = 4; 20 nM siRNA) by TNF-α activated bEnd.3 cells in the absence or the presence of excess of P-selectin binding peptide (Psel_BP), observed by fluorescence microscopy, at different time interval in static conditions of incubation [lipoplexes (**red**) and cell nuclei (**blue**); bar 50 μm]. (**B**) Quantification of lipoplexes uptake by the cells in static conditions expressed as ratio of red pixels percentage to blue pixels percentage for each image field (each point represent media of 9 fields). Bar graph shows data as mean ± S.E.M. *** p < 0.0001. (**C**) Time-lapse observation of dynamic interaction between Rhodamine B-labelled lipoplexes (Psel-lipo/siRNA and Scr-lipo/siRNA) and bEnd.3 cells in FCS2 system using an inverted fluorescence microscope. Overlay of phase contrast and lipoplexes fluorescence images (**red**) is presented (bar 200 μm) and of lipoplexes fluorescence (Rhodamine-PE; **red**) and nuclei fluorescence (DAPI; **blue**) (bar 50 μm).

The fluorescence images obtained in the case of incubation in static conditions revealed that Psel-lipo/siRNA lipoplexes were internalized to a significantly higher extent by TNF-α activated bEnd.3 cells in comparison to Scr-lipo/siRNA lipoplexes at all time points investigated (Figure 5A). The quantification of the global association (binding and uptake) of lipoplexes with the cells was done by calculating the red pixels originating from Rhodamine-labelled Psel-lipo/siRNA and Scr-lipo/siRNA lipoplexes that were normalized to the blue pixels (corresponding to DAPI stained nuclei) for each image field (Figure 5B). 

It can be observed that the global association of Psel-lipo/siRNA lipoplexes with b.End3 cells was significantly higher as compared to non-targeted Scr-lipo/siRNA at the same time points (10, 30, 60 and 240 minutes). To assess the specificity of Psel-lipo/siRNA association with TNF-α activated EC, the cells were preincubated with an excess of free P-selectin binding peptide before incubation with P-selectin targeted lipoplexes (Figure 5A,B). This condition significantly impaired the association of Psel-lipo/siRNA with activated EC, the percentages of inhibition being ~52% at 10 minutes, ~62% at 30 minutes, ~65% at 60 minutes and ~68% at 240 minutes (Figure 5B). This result indicated and confirmed that the binding and uptake of Psel-lipo/siRNA by TNF-α activated EC takes place via a P-selectin specific mechanism, mediated by the affinity peptide coupled on the surface of cationic liposomes. 

Likewise, the experiments performed in flow conditions were in line with the experiments done in static conditions and revealed that the uptake of lipoplexes by TNF-α activated bEnd.3 cells was higher for Psel-lipo/siRNA lipoplexes compared with Scr-lipo/siRNA lipoplexes (Figure 5C).

#### 3.4.3. Psel-Lipo/siRNA Lipoplexes Efficiently Deliver siRNA into TNF-α Activated Endothelial Cells

**Figure 6:**
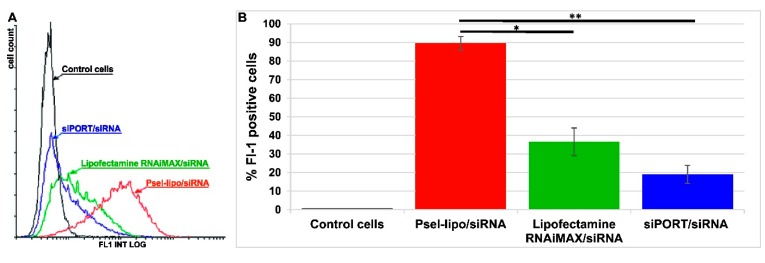
(**A**) Flow cytometry histogram overlay showing FITC-positive bEnd.3 cells after incubation with Psel-lipo/FITC-siRNA lipoplexes (charge ratio R +/− = 4; 20 nM siRNA), Lipofectamine™ RNAiMAX/FITC-siRNA and siPORT™/FITC-siRNA. (**B**) Flow cytometry bar graph shows data as mean ± S.E.M. (representative experiment performed in triplicate) * p < 0.05, ** p < 0.01.

The capacity of Psel-lipo/siRNA lipoplexes to transfer siRNA into activated EC was established by determination of the uptake of FITC-labelled control siRNA in comparison with other commercially available vectors for siRNA delivery. The flow cytometry results showed that, after 4 hours of incubation with Psel-lipo/FITC-siRNA lipoplexes (R+/− = 4; 20 nM siRNA), almost 90% of the cells were FITC-positive, while Lipofectamine™ RNAi MAX /FITC-siRNA and siPORTTM NeoFX/FITC-siRNA resulted into just ~40% and ~20% fluorescent cells (Figure 6). Moreover, the average intensity of fluorescence is significantly greater for Psel-lipo/FITC-siRNA lipoplexes as compared with the other vectors, suggesting that the average amount of internalized siRNA is larger (Figure 6A).

### 3.5. Psel-Lipo/siRNA-GAPDH Lipoplexes Knock-Down the Expression of GAPDH mRNA in Endothelial Cells

**Figure 7:**
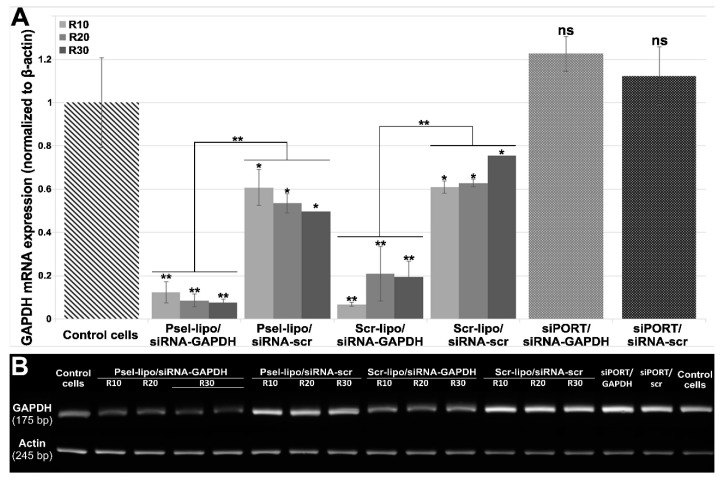
(**A**) The expression of GAPDH mRNA in b.End3 cells was significantly reduced by Psel-lipo/siRNA-GAPDH (100 nM siRNA). GAPDH mRNA expression was determined by quantitative RT-PCR at 48 hours after transfection using different vectors. The relative levels of GAPDH mRNA were normalized using β-actin as an internal control. (**B**) Agarose gel electrophoresis run with amplicons obtained after real-time PCR reactions. The data are representative of three independent experiments. ** p < 0.01, * p < 0.05, ns: non-significant versus control cells; ** p < 0.01 Psel-lipo/siRNA-GAPDH versus Psel-lipo/siRNA-scr and Scr-lipo/siRNA-GAPDH versus Scr-lipo/siRNA-scr.

To evaluate whether Psel-lipo/siRNA lipoplexes are efficient in down-regulation of a target mRNA in EC, we used a specific siRNA targeting the ubiquitous metabolic enzyme glyceraldehyde-3-phosphate dehydrogenase (GAPDH), a housekeeping gene. Two types of lipoplexes (at three charge ratios +/− of 4, 10, 20 and 30), either P-selectin targeted (Psel-lipo/siRNA) or non-targeted (Scr-lipo/siRNA), were used to deliver 100 nM siRNA-GAPDH or a scrambled-siRNA sequence into b.End3 cells. Since, GAPDH show high expression levels in cells we chose 100 nM siRNA in order to obtain a greater reduction in its mRNA level. At a charge ratio +/− of 4 we have not obtained a GAPDH downregulation (Supplementary Figure S1). In Figure 7A, GAPDH mRNA levels relative to β-actin are presented as fold change as compared to the control cells (unsilenced condition), using the 2^−ΔΔCT^ method. It can be seen that Psel-lipo/ siRNA-GAPDH significantly knockdown GAPDH mRNA level (by ~90%) at charge ratios of 10, 20 and 30. Also, a down-regulation by approx. 80–90% of GAPDH mRNA expression was obtained in the case of transfection using lipoplexes formed between non-targeted liposomes (Scr-lipo) and siRNA-GAPDH. In the case of transfection with a scrambled siRNA duplex using both types of nanocarriers, non-targeted or P-selectin targeted, a reduction of 25% to 50% of GAPDH mRNA level normalized to beta-actin was obtained. One possible explanation of the reduced GAPDH mRNA level expressed relative to that of β-actin, observed in the case of treating the cells with lipoplexes carrying scrambled siRNA, may be the changes in the expression of β-actin gene induced by the experimental conditions, as previously reported by others ([40]). When the siRNA-GAPDH transfection was done using the commercial vector siPORT^TM^ using the manufacturer’s instructions, the mRNA GAPDH level was not significantly reduced. This unexpected result may be explained by the reduced cellular uptake of FITC-labelled irrelevant siRNA delivered in EC using siPORT^TM^ NeoFX vector in comparison with delivery of siRNA using P-sel-lipo/FITC-siRNA lipoplexes. At the end of real-time PCR reaction, after 40 cycles, the PCR reaction products were run on an agarose gel (Figure 7B). It can be observed that the bands corresponding to the transfection with Psel-lipo/siRNA GAPDH are much weaker than that observed for transfection with Scr-lipo/siRNA-GAPDH or with siRNA-scrambled delivered by P-selectin targeted or non-targeted lipoplexes.

## 4. Discussions

Due to the crucial role played in the onset and maintaining of vascular inflammation, the endothelium is an attractive target for pharmacological intervention. Moreover, the endothelium targeting has the advantage that it is reachable for intravenously administered drugs. RNA interference (RNAi) may represent an appropriate therapeutic method to calm down the inflamed endothelium by post-transcriptional gene silencing of major players involved in the continuous exacerbation of the inflammatory response (e.g. transcription factors, signalling kinases, cytokines) [41].

Certainly, progress towards the clinical use of siRNA therapeutics depends on the development of suitable siRNA carriers [42,43]. There were several attempts to develop proper nanocarriers, such as liposomes for specific and efficient siRNA delivery to endothelium in vitro and in vivo [44]. There are many challenges in the development of efficacious lipid-based siRNA nanocarriers such as the appropriate composition of liposomes, the ratio of cationic lipid to nucleic acid used for lipoplexes formation, protection of siRNA against degradation, optimal concentration of either siRNA and liposomes, cytotoxicity of lipoplexes, efficient cellular uptake and effective endosomal release. The cell adhesion molecule P-selectin can be used as target for nanocarriers because of its strong presence on the membrane of activated EC in both acute [45] and chronic inflammation [16]. Also, it was reported that the internalization of P-selectin by EC is very efficient employing endosomes, trans-Golgi network (TGN) and the storage granules [19,20] and thus its use as target for nanotherapy has the benefit of employing an intracellular route that circumvents the lysosomal degradation of the therapeutic agents entrapped into nanoparticles. 

We have previously reported that lipid nanoemulsions surfaced with peptide with high affinity for P-selectin bind and are internalized by TNF-α activated endothelial cells and their drug cargo (dexamethasone) reduces mRNA levels of pro-inflammatory cytokines in these cells and decrease monocytes adhesion and transmigration across EC [27]. Also, work from another group showed that polystyrene nanoparticles targeted to P-selectin by conjugating glycocalicin, the extracellular segment of platelet glycoprotein (GP) Ibα, onto the surface exhibit higher cellular uptake by activated human aortic EC under physiological flow conditions as compared to control, non-targeted nanoparticles [26]. 

In this study, we show that P-selectin can be used as a target to specifically deliver siRNA to activated EC using PEGylated cationic liposomes. In our experiments, P-selectin was detected on the surface of immortalized mouse brain endothelial b.End3 cells, in the absence of activation. However, the treatment for 4 hours with TNF-α causes an about two-fold increase of P-selectin level at the cell surface. This result is in good agreement with previous studies reporting that TNF-α induces P-selectin synthesis in mouse endothelioma cells [5] and leads to functionally detectable P-selectin at the cell surface [46]. Thus, we developed and characterized P-selectin targeted lipoplexes formed by complexation of siRNA with P-selectin targeted PEGylated cationic liposomes comprising DMAPAP and DOPE (50:49 mol%) at different charge ratios R +/−. Cationic liposomes containing DMAPAP lipid have been successfully used previously to deliver siRNA to silence TNF-α in experimental arthritis [36] and Receptor Activator of Nuclear factor Kappa-B (RANK) in a polyethylene particle-induced osteolysis model [47]. 

In this study, to endow specificity for activated EC, a peptide with affinity for P-selectin was covalently coupled to the distal ends of PEGylated phospholipid anchor inserted in the membrane of liposomes (Psel-lipo). The mean hydrodynamic diameter and ζ-potential were similar for both P-selectin targeted and non-targeted lipoplexes (Psel-lipo/siRNA and Scr-lipo/siRNA) and were around 400 nm and above +30 mV, respectively for charge ratios +/− of 10, 20 and 30. The ability of Psel-lipo/siRNA to pack and protect siRNA was determined by agarose gel retardation assay. Our data showed that Psel-lipo/siRNA was able to completely impede the migration of siRNA in agarose gel starting with charge ratio of 4 and to protect the siRNA, that was released from complexes after addition of TritonX-100 surfactant. 

Next, the interaction of P-selectin targeted lipoplexes with activated EC was investigated in both static and flow chamber experiments. Since endothelial cells are continuously exposed to flow conditions, investigating nanoparticle-endothelial interactions under flow is needed to estimate the cell responses in physiological-relevant environments. The global cellular association (binding and internalization) of Psel-lipo/siRNA with activated EC was significantly enhanced as compared with control, cationic liposomes coupled with a scrambled peptide (Scr-lipo/siRNA). Moreover, the competitive studies using an excess of free peptide with P-selectin affinity revealed the specificity of P-selectin mediated mechanism of uptake. 

Psel-lipo/siRNA was efficiently internalized as demonstrated by fluorescence microscopy that evidenced intracellular fluorescent dots around nuclei, suggesting a cytoplasmic localization of targeted lipoplexes. Using a FITC-labelled siRNA we provide data that show an efficient intracellular delivery of siRNA into activated EC mediated by Psel-lipo/siRNA-FITC lipoplexes (90% of cells positive). As compared, commercial vectors optimized for siRNA transfection determined a significantly lower level of intracellular siRNA-FITC fluorescence.

To test whether intracellularly delivered siRNA is functional, we investigated if a siRNA sequence targeting the mouse GAPDH transcript delivered into EC via P-selectin lipoplexes (Psel-lipo/siRNA-GAPDH) induces silencing of GAPDH mRNA expression. As control for specificity, a scrambled siRNA designed to not recognize any mammalian mRNA sequence was used. Since we have not obtained a knock-down of GAPDH with lipoplexes formed with siRNA-GAPDH at charge ratio +/− of 4, we increased the charge ratio to 10, 20 and 30 in order to promote transfection efficiency in vitro. At 48 hours after transfection of EC with Psel-lipo/siRNA-GAPDH (charge ratios +/− of 10, 20 and 30), over 90% inhibition of GAPDH mRNA (normalized to β-actin levels) was detected. Also, in the case of transfection with Scr-lipo/siRNA-GAPDH lipoplexes, a similar percentage of down-regulation in GAPDH mRNA level was obtained. One possible explanation of the fact that there is no difference between targeted and non-targeted lipoplexes at +/− charge ratios of 10, 20 and 30 might be the high positive values of zeta potential that may determine non-specific cellular uptake. Also, the result could be explained by the fact that the cells were continuously incubated with lipoplexes for 48 hours, therefore even if initially, in the first 4 hours, the uptake of Psel-lipo/siRNA lipoplexes is much higher than that of Scr-lipo/siRNA lipoplexes, after 48 hours the level of intracellularly delivered siRNA by both types of lipoplexes produces the same degree of GAPDH knock-down. Our data are in line with those obtained by Ásgeirsdóttir et al. 2009 [48] showing no difference between E-selectin targeted and non-targeted transfection, when a high concentration of siRNA (60 nM) is used for VE-cadherin silencing in activated glomerular EC. A certain degree of down-regulation (between 25% and 50%) of GAPDH mRNA relative to β-actin was obtained when transfection was done with a scrambled siRNA duplex using both type of nanocarriers, either P-selectin targeted or non-targeted, suggesting off-target effects of scrambled siRNA. In addition, the unspecific downregulation of GAPDH by scrambled siRNA could be due to the liposomes’ composition or to the variability of the RT-PCR assay and the variation in the expression of β-actin gene in different experimental conditions.

These P-selectin targeted siRNA nanocarriers developed by us showed their efficiency in vitro. It remains to be tested whether they function in vivo after intravenous administration. Different charge ratios +/− have to be tested in vivo to establish the optimal ratio that allow both, a specific binding to activated endothelium expressing P-selectin and the highest transfection efficiency in endothelial cells. There are data to believe that the presence of PEG2000 on the surface of cationic liposomes can contribute to the increased survival of targeted lipoplexes into circulation by reducing their non-specific association with plasma components and the uptake by the cells of mononuclear phagocytic system [49]. Since P-selectin is expressed by both platelets and activated endothelium, one can assume that the nanoparticles targeted to P-selectin are not entirely specific to activated endothelial cells [50]. However, our previous experiments showed that fluorescently labelled P-selectin targeted lipid nanoemulsions do not bind specifically to platelets of i.v. LPS-treated mice [27].

The results obtained in this study demonstrate an effective targeted delivery of siRNA into cultured activated endothelial cells. This proof of concept motivates further examination of in vivo delivery of targeted Psel-lipo/siRNA lipoplexes to activated endothelium for specific silencing the genes responsible for progression and exacerbation of chronic inflammation in atherosclerosis and in other various pathologies.

## 5. Conclusions

We developed siRNA nanocarriers targeted to a cell adhesion molecule, P-selectin, that are able to efficiently protect the encapsulated siRNA from exogenous factors. These targeted siRNA nanocarriers bind specifically to TNF-α activated endothelial cells, deliver with high efficiency siRNA into the target cells thus leading to specific silencing of the chosen gene.

## Supplementary Materials

The following are available online at http://www.mdpi.com/1999-4923/11/1/47/s1, Figure S1: The expression of GAPDH and β-actin mRNA in b.End3 cells determined at 48 hours after transfection with different lipoplexes at a charge ratio +/− of 4 and 100 nM siRNA. Legend: Scr-lipo: non-targeted liposomes; Psel-lipo: P-selectin targeted liposomes; scr-siRNA: scrambled siRNA; siRNA-GAPDH: siRNA specific for GAPDH; siPORT: siPORTTM NeoFX transfection vector.
