# Supplementary material for: Targeted Transfection Using PEGylated Cationic Liposomes Directed Towards P-Selectin Increases siRNA Delivery into Activated Endothelial Cells
Source: Pharmaceutics. 2019 Jan 21;11(1):47. doi: 10.3390/pharmaceutics11010047 (PMC6359248; doi:10.3390/pharmaceutics11010047)
Supplement: Supplementary file 1 [file pharmaceutics-11-00047-s001.pdf]

pharmaceutics

MDPI

Targeted Transfection Using PEGylated Cationic Liposomes Directed Towards P-Selectin Increases siRNA Delivery into Activated Endothelial Cells

Cristina Ana Constantinescu [1,2], Elena Valeria Fuior [1], Daniela Rebleanu [1], Mariana Deleanu [1,3], Viorel Simion [1], Geanina Voicu [1], Virginie Escriou [4,5,6,7], Ileana Manduteanu [1], Maya Simionescu [1] and Manuela Calin [1,*]

1 Institute of Cellular Biology and Pathology "Nicolae Simionescu," Bucharest 050568, Romania; cristina.constantinescu@icbp.ro (C.A.C.); elena.fuior@icbp.ro (E.V.F.); daniela.rebleanu@icbp.ro (D.R.); mariana.deleanu@icbp.ro (M.D.); viorel.simion@icbp.ro (V.S.); geanina.voicu@icbp.ro (G.V.); ileana.manduteanu@icbp.ro (I.M.); maya.simionescu@icbp.ro (M.S.)

2 University of Agronomic Sciences and Veterinary Medicine (UASVM), Faculty of Veterinary Medicine, Bucharest 050097, Romania

3 University of Agronomic Sciences and Veterinary Medicine (UASVM), Faculty of Biotechnologies, Bucharest 011464, Romania

4 Centre National de la Recherche Scientifique (CNRS), Unité de Technologies Chimiques et Biologiques pour la Santé (UTCBS) UMR 8258, Paris 75006, France; virginie.escriou@parisdecartes.fr

5 Institut National de la Santé et de la Recherche Médicale (INSERM), Unité de Technologies Chimiques et Biologiques pour la Santé (UTCBS) U 1022, Paris 75006, France;

6 Université Paris Descartes, Sorbonne-Paris-Cité University, Unité de Technologies Chimiques et Biologiques pour la Santé (UTCBS), Paris 75006, France

7 Chimie ParisTech, PSL Research University, UTCBS, Paris 75005, France

* Correspondence: manuela.calin@icbp.ro; Tel: +4021-319-45-18; Fax: +4021-319-45-19

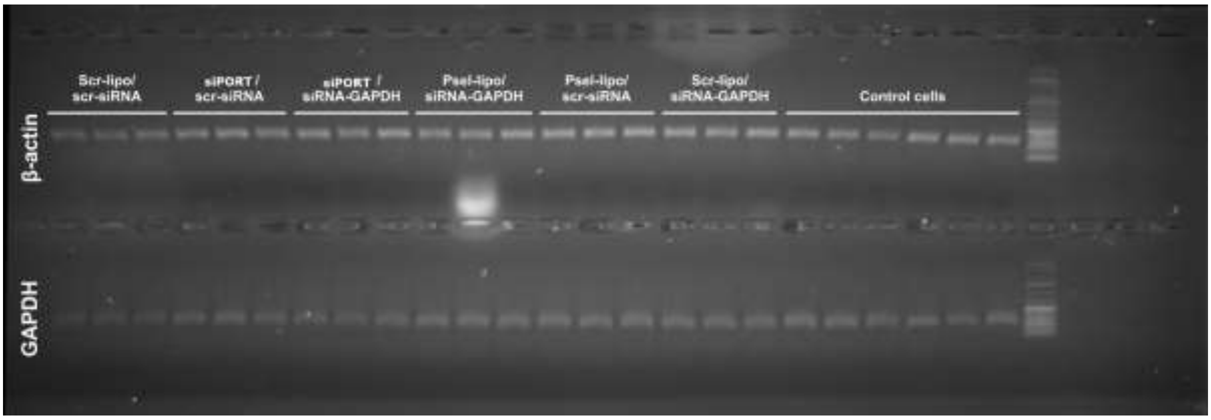

Supplementary Figure 1. The expression of GAPDH and β-actin mRNA in b.End3 cells determined at 48 hours after transfection with different lipoplexes at a charge ratio +/- of 4 and 100 nM siRNA. Legend: Scr-lipo: non-targeted liposomes; Psel-lipo: P-selectin targeted liposomes; scr-siRNA: scrambled siRNA; siRNA-GAPDH: siRNA specific for GAPDH; siPORT: siPORT™ NeoFX transfection vector.
